# Discrimination of bovine milk from non-dairy milk by lipids fingerprinting using routine matrix-assisted laser desorption ionization mass spectrometry

**DOI:** 10.1038/s41598-020-62113-9

**Published:** 2020-03-20

**Authors:** Philippa England, Wenhao Tang, Markus Kostrzewa, Vahid Shahrezaei, Gerald Larrouy-Maumus

**Affiliations:** 1 MRC Centre for Molecular Bacteriology and Infection, Department of Life Sciences, Faculty of Natural Sciences, Imperial College London, London, SW7 2AZ UK; 2 Department of Mathematics, Imperial College London, London, United Kingdom; 3 Bruker Daltonik GmbH, Bremen, Germany

**Keywords:** Lipidomics, Assay systems

## Abstract

An important sustainable development goal for any country is to ensure food security by producing a sufficient and safe food supply. This is the case for bovine milk where addition of non-dairy milks such as vegetables (e.g., soya or coconut) has become a common source of adulteration and fraud. Conventionally, gas chromatography techniques are used to detect key lipids (e.g., triacylglycerols) has an effective read-out of assessing milks origins and to detect foreign milks in bovine milks. However, such approach requires several sample preparation steps and a dedicated laboratory environment, precluding a high throughput process. To cope with this need, here, we aimed to develop a novel and simple method without organic solvent extractions for the detection of bovine and non-dairy milks based on lipids fingerprint by routine MALDI-TOF mass spectrometry (MS). The optimized method relies on the simple dilution of milks in water followed by MALDI-TOF MS analyses in the positive linear ion mode and using a matrix consisting of a 9:1 mixture of 2,5-dihydroxybenzoic acid and 2-hydroxy-5-methoxybenzoic acid (super-DHB) solubilized at 10 mg/mL in 70% ethanol. This sensitive, inexpensive, and rapid method has potential for use in food authenticity applications.

## Introduction

Today, humanity is facing many challenges globally, which are primarily driven by our rapidly growing world population. A major consequence of this is the rise in the global demand for food, which runs in parallel with increased competition for resources (e.g., land and water), therefore a huge pressure has built up on the food production industry. To make matters worse, climate change and globalization are contributing to the spread of pathogens, generating a host of new problems such as an increased uncertainty in the food supply. An important sustainable development goal for any country is to ensure agri-food security by producing a sufficient and safe food supply. Such global challenges must be addressed through the implementation of novel technologies as we continue to enhance our biological understanding of food science. As for example, food related illness costs the UK health service approximately £6Bn annually^1^. Therefore, to achieve food security we must sustainably produce a safe and adequate food supply.

Particularly, the high nutritional value of dairy products, the demand and supply gap, the perishable nature of milk and lack of suitable detection tests are all potential reasons as to why the dairy industry is increasingly subjected to food fraud across the world^2^. These frauds then have a knock-on effect to the price of these products, hence the motivation behind food fraud is economic, but the impact is a real concern to public health globally. Bovine milk is the most common adulterated milk. The addition of non-dairy milks to dairy products and the opposite dairy milk in non-dairy milks is an old and illegal practice in several countries and has become highly common and complex^3,4^. This may not seem to be a major problem; however, such adulteration can have severe sanitary and ethical consequences as it will cause consumers to be exposed to hidden allergens found in bovine milk such as the proteins αS1-casein, αS2-casein, β-casein, κ-casein, α-lactalbumin, β- lactoglobulin, immunoglobulins, bovine serum albumin and traces of lactoferrin leading to symptoms ranging from hives and itches to anaphylaxis caused by a large increase in immunoglobulin E (IgE) antibodies^5,6^. Similarly, soy allergy is very common and can have dramatic consequences as the ones found with bovine milk^7–9^.

Therefore, sensitive, rapid and cost-effective detection techniques must be employed in order to prevent such fraud and improve food safety.

Currently, the techniques in place include PCR^10^, spectroscopy technics^11,12^ protein analysis by liquid chromatography combined with mass spectrometry^13^, proteins and lipids analysis by MALDI-TOF MS^14–21^. As for example, the recent literature present the detection of adulterated plant proteins in raw milk by UPLC-quadrupole time-of-flight mass spectrometric proteomics^22–24^. Despite being promising and powerful as such approaches are able to detect adulteration as low as 1% (wt/wt), the sample preparations and data processing still remain challenging for the day-to-day routine.

In addition to those technics, lipids and fatty acids composition are being used to detect milk adulteration as non-dairy milks possess longer fatty acid chain than dairy milk allowing their use as markers of adulteration^3,25,26^. In this approach and based on this knowledge, the sample preparation is composed of several steps, including extraction, saponification and derivatization, prior analysis of the derivatized fatty acids, usually from triacylglycerols (TAGs), by gas chromatography^4,27–29^. In parallel to gas chromatography, due to its ease to use and the limited sample preparation, MALDI-TOF is seen as valuable tool for the analysis of dairy milk adulteration. For example, Calvano and colleagues used MALDI-TOF for the determination of phospholipids in order to detect lipid markers of cows’ milk in sheep’s and in goats’ milk^16,17^. Despite been able to discriminate among the milk tested, the main drawback of the study is the use of lipid enrichment methods such as Bligh and Dyer extraction when carrying out MALDI-TOF MS, which cannot be high-throughput and deals with large amount of highly toxic organic solvents such as chloroform and methanol, which required dedicated laboratory.

In addition, to the best of our knowledge, there are very limited researches on the authentication of bovine milk and non-dairy milks by using routine MALDI-TOF MS to date, equipment already used to identify food pathogens and therefore available in many laboratories for food-testing. We believe that the different biomarkers components between bovine milk and non-dairy milks could be very informative and provide a clear distinction between the sources of the milks.

Here, to address this gap in milks discrimination and adulteration detection, we compared bovine milk with soya milk and coconut milk as the two sources of plant-based milks, which are two of the most common substitutes or alternatives for bovine milk^30–32^. For example, soya milk is a common vegan alternative to bovine milk^9,33–35^ and therefore adulteration can be attractive to fraud. Due to the similar properties to bovine milk, soya milk has been found to be added to bovine milk for revenue maximization^36,37^. In addition, plant derivatives, such as oils and proteins, is a potential candidate to spike bovine milk products for economic reasons^38,39^ but can cause serious food safety incidents as mentioned earlier via the presence of hidden allergens^7–9,40^. Coconut milk or juice have become the must-have drink for the heath-conscious despite higher high-street price than bovine milk which can be attractive to fraud^36,39^ and that might lead to disastrous health consequences^41^. To illustrate that, in January 2016, the UK Food Standards Agency (FSA) issued warnings towards the presence of undeclared milk, such as cow’s milk, in coconut drinks following on the Australia’s department of agriculture to test for foreign additives in coconut milk as a result of a reported case of the death of a 10-year-boy from an allergic reaction to coconut drink^42^. Regarding bovine milks, according to the literature, there are some evidence of differences in the composition between organic and conventional dairy milks^43–47^. For example, organic dairy products seem to contain higher protein, and total omega-3 fatty acid, cis-9, trans-11 conjugated linoleic acid, trans-11 vaccenic acid, eicosapentanoic acid, and docosapentanoic acid compared to conventional dairy products. That is why, in this study, we used organic and conventional bovine milk to test if the new developed methodology is also able to discriminate those two groups in addition to discriminate between bovine milks from non-dairy milks.

Here, we have developed a workflow for the identification and discrimination of bovine milk from non-dairy milks as well as the detection of milk adulteration based on a one-step lipid fingerprint using routine MALDI-TOF mass spectrometer allowing to a simplify analysis within the agri-food environment.

## Material and Methods

### Materials

Commercial milks were purchased from local supermarkets from the period of April 2019 to May 2019 and from January 2020 to February 2020. They were analyzed as close to the day of purchase as possible. The milks tested included four types: organic bovine milk (n = 12), bovine milk (n = 12), coconut milk (n = 12) and soya milk (n = 12). Milks were aliquoted in 1 mL aliquots and were stored at 4 °C prior analysis by MALDI-TOF or −80 °C for longer period of storage. To detect adulteration, experiments were performed as follow: bovine milk 100%, bovine milk 95% + non-dairy milk 5%, bovine milk 90% + non-dairy milk 10%, bovine milk 80% + non-dairy milk 20%, bovine milk 70% + non-dairy milk 30%, bovine milk 60% + non-dairy milk 40%, bovine milk 50% + non-dairy milk 50%, bovine milk 40% + non-dairy milk 60%, bovine milk 30% + non-dairy milk 70%, bovine milk 20% + non-dairy milk 80%, bovine milk 10% + non-dairy milk 90% and non-dairy milk 100%. The samples were mixed, vortexed for 30 seconds at room temperature and prepared as descried below.

### Sample preparation

Milk were taken straight from the carton into an Eppendorf tube. 4 technical replicates were performed per sample. We optimized the dilution of the milk samples into double distilled water prior to MS analysis in order to get the best signal-to-noise ratio and mass resolution. We found that milk diluted 1:4 with double-distilled water was most appropriate for our experiments. The matrix used consists of a 9:1 mixture of 2,5-dihydroxybenzoic acid and 2-hydroxy-5-methoxybenzoic acid (super-DHB, Sigma-Aldrich) at a concentration of 10 mg/mL in 70% ethanol, and 1.2 µL of this was loaded onto 0.4 µl of 1:4 diluted milk sample. Additionally, for external calibration, 0.5 µL of calibration peptide was loaded along with 0.5 µL of the given calibration matrix (peptide calibration standard II, Bruker Daltonik, Germany). The samples were loaded onto a disposable MBT 96 Biotarget plate (Bruker Part-No. 1840375).

### Mass spectrometry analysis

MS analyses were performed on a MALDI Biotyper Sirius system (Bruker Daltonik, Germany). The mass spectra were scanned in the range of *m*/*z* 400 to 1,000. The mass profiles were acquired using FlexControl 3.4 software (Bruker Daltonik, Germany). The spectra were recorded in the linear positive-ion mode (laser intensity 95%, ion source 1 = 10.00 kV, ion source 2 = 8.98 kV, lens = 3.00 kV, detector voltage = 2652 V, pulsed ion extraction = 150 ns). Each spectrum corresponded to ion accumulation of 5,000 laser shots randomly distributed on the spot. The spectra obtained were processed with default parameters using FlexAnalysis v.3.4 software (Bruker Daltonik, Germany).

Assignments were based on the MS/MS fragmentation profile acquired on a 4800 Proteomics Analyzer (with TOF-TOF Optics, Applied Biosystems, plate: 384 Opti-TOF 123 mm × 84 mm AB Sciex NC0318050, 1016629) using the reflectron mode. Samples were analyzed operating at 20 kV in the positive ion mode. MS/MS mass spectrometry data were analyzed using Data Explorer version 4.9 from Applied Biosystems.

### Statistical analysis

Data pre-processing and visualizations were achieved as previously described^48,49^. For milk adulteration detection performance, quadratic regression was applied to fit the model:

$$\log (y)=a{x}^{2}+bx+c$$

here *x* stands for the percentage of non-dairy milk that had been added (unit: %), and log(*y*) stands for the logarithm of ratio of the relative abundances.

### Ethical approval

This article does not contain any studies with animals performed by any of the authors.

## Results and Discussion

**Figure 1:**
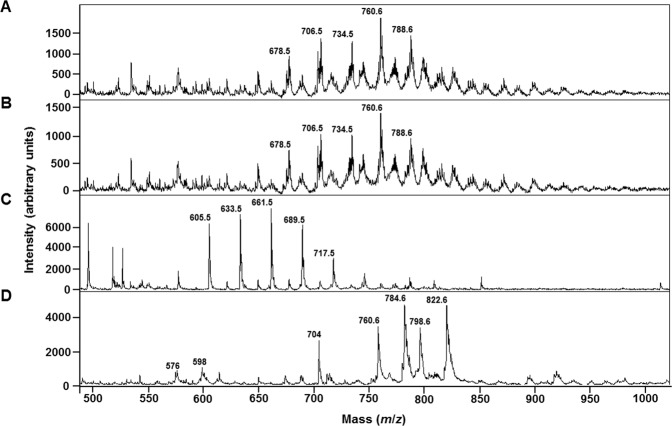
MALDI-TOF linear positive-ion mode mass spectra of organic bovine milk (**A**), whole bovine milk (**B**), coconut milk (**C**) and soya milk (**D**). Spectra were acquired using super-DHB as matrix solubilized at 10 mg/mL in 70% ethanol.

In this study, we first optimized the dilution of the milk samples prior to MS analyses in the positive ion mode. We found that milk diluted 1:4 with double-distilled water was most appropriate for our experiments. This was decided following observation of the raw mass spectra for the four types of milk at six different levels of dilution: undiluted, 1:2, 1:4, 1:6, 1:10 and 1:20 in double-distilled water (data not shown). Although 1:6 and 1:10 dilution gave acceptable mass spectra, the 1:4 dilution gave spectra with the highest signal-to-noise (S/N) (>10) and mass resolution (>200) (Fig. S1). The fact that undiluted and 1:2 diluted mild did not generate spectra could be explained by the poor co-crystallization with the matrix leading to a viscous spot on the MALDI target plate precluding any transfer of the energy from the laser to the matrix in order enable the desorption of the molecules of interest^50,51^. Apart optimizing the dilution of the milk, we also tested different solvent mixtures (chloroform, methanol, and ethanol) to solubilize the matrix in order to generate high-quality and reproducible mass spectra. After numerous iterations of optimization, the matrix solvent composed of 70% ethanol gave mass spectra with a S/N greater than 10 (Fig. S1). Due to its versatility for the analysis of phospholipids, we chose to use super-DHB as matrix^52^. Lipid assignments were based on the MS/MS profiles (Supplementary Figs. S2–S4, and Supplementary Table S1), the use of LIPID MAPS database (http://www.lipidmaps.org/) and literature data^14,16,53,54^. In both bovine and soya milks, the most abundant peaks present in all the samples were those assigned to phosphatidylcholine (PC). In coconut milk, the most abundant peaks present in all the samples were those of triacylglycerols (TAGs). The spectra of bovine milk are dominated by a set of peaks at *m*/*z* 678.5, *m*/*z* 706.5, *m*/*z* 734.5, *m*/*z* 760.5 and *m*/*z* 788.5 assigned to the [M+H]+ molecular ions of PC(28:0), PC(30:0), PC(32:0), PC(34:1) and PC(36:1), respectively (Fig. 1A,B). The mass spectrum profile obtained for coconut milk is dominated by a set of peaks at *m*/*z* 605.5, *m*/*z* 633.5, *m*/*z* 661.5, *m*/*z* 689.5 and *m*/*z* 717.5 assigned to the [M+H]+ molecular ions of TAG(32:0), TAG(34:0), TAG(36:0), TAG(38:0) and TAG(40:0), respectively (Fig. 1C). The mass spectrum profile obtained for soya milk is dominated by a set of peaks at *m*/*z* 760.6, *m*/*z* 784.6, *m*/*z* 798.6 and *m*/*z* 822.6 assigned to the [M+H]+ molecular ions of PC(34:1), PC(36:3), PC(37:3) and PC(39:5), respectively (Fig. 1D). As seen in the mass spectra, while organic and non-organic bovine milks gave similar profile, the data obtained clearly show that our simplified method generate a unique fingerprint of the different milk types tested here.

**Figure 2:**
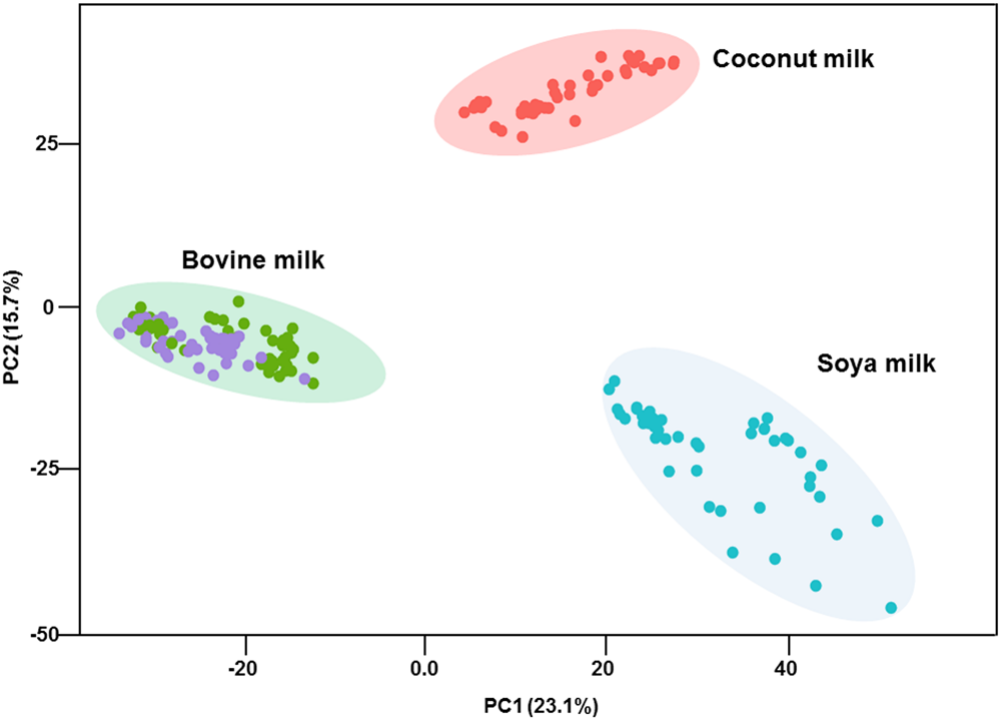
PCA of the MALDI-TOF MS milk data (n = 12 samples and 4 technical replicates per samples). Green and violet correspond to non-organic and organic bovine milk respectively. Coconut milk is displayed in red and soya milk in blue.

To test statistically the performance of the one-step lipids MALDI-TOF MS fingerprint, we performed Principal Component Analysis (PCA). Data pre-processing and visualizations were achieved as previously described^48,49^. During the preprocessing, instead of doing alignment across samples, we combined the intensities across samples and then use the function “removeBatchEffect” from the R package “limma” to correct batch effect^55^. Figure 2 shows the scatter plot of PC1 versus PC2 for the MS data of the milks tested in this study. Although no discrimination was noticed between organic and conventional bovine milk groups, this new methodology showed that the type of milks (bovine, coconut, and soya) were clearly grouped and separated.

**Figure 3:**
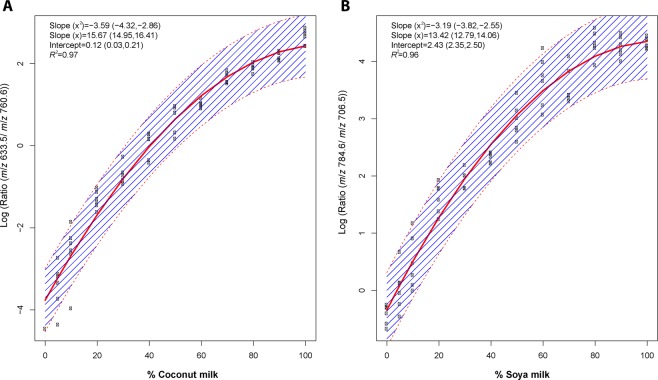
Plot of the ratios of the relative abundances of the marker ions at *m*/*z* 633.5 and *m*/*z* 760.6 for bovine milk in coconut milk against the percentage of coconut milk (**A**). Plot of the ratios of the relative abundances of the marker ions at *m*/*z* 784.6 and *m*/*z* 706.5 for bovine milk in soya milk against the percentage of soya milk (**B**). Experiments were done in 6 biological replicates. Numbers in the brackets indicate the 95% confidence interval of the estimates. The bands between the dashed lines stand for the 95% confidence interval of the predictions. The red solid line is the fitted line. Experiment was performed in 6 technical replicates.

To test the hypothesis that the signature found earlier could serve as a read-out of milk adulteration, binary mixtures containing bovine milk and coconut milk and, bovine milk and soya milk were prepared, subjected to the sample preparation described in the material and methods section and analyzed by routine MALDI-TOF MS. The MALDI spectra obtained from the bovine and non-dairy milks adulterated at 5%, 10%, 20%, 30%, 40%, 50%, 60%, 70%, 80%, and 90% (Figs. S5, S6). In the spectra obtained from coconut milk adulteration by bovine milk, the relative abundance of the ion at *m*/*z* 633.5 gradually increase passing from 0% to 100% coconut milk. Similar to that observation, in the spectra obtained from soya milk adulteration by bovine milk, the relative abundance of the ion at *m*/*z* 784.6 progressively increase passing from 0% to 100% soya milk. Therefore, the ratio of the relative abundances of the ions at *m*/*z* 633.5, attributed to coconut milk, and *m*/*z* 760.6, attributed to bovine milk, can be used to detect bovine milk adulteration in coconut milk. Similarly, the ratio of the relative abundances of the ions at *m*/*z* 784.6, attributed to soya milk, and *m*/*z* 706.5, attributed to bovine milk, can be used to detect bovine milk adulteration in soya milk. Therefore, the level of adulteration can be determined as a concentration-dependent relationship exists between the relative intensities of the lipid markers identified (Figs. S5, S6). This observation was confirmed by plotting the logarithm of the ratio of the relative abundances of the peaks (*m*/*z* 633.5/ *m*/*z* 760.6) and (*m*/*z* 784.6/ *m*/*z* 706.5) against the percentage of adulterant bovine milk in coconut milk and bovine milk in soya milk, respectively (Fig. 3A,B).

**Figure 4:**
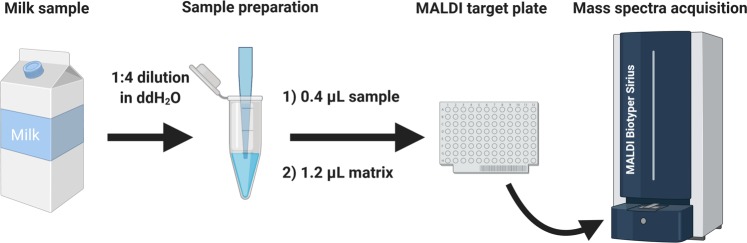
Schematic diagram of the sample preparation process for the test performed on the MALDI Biotyper Sirius system (Bruker Daltonics). Milk samples are first diluted 1:4 in double-distilled H_2_O (ddH_2_O). 0.4 μL of this preparation are loaded into the MALDI Biotarget plate followed by the addition of 1.2 μL of the matrix (super-DHB solubilized at 10 mg/mL in 70% ethanol) and mixed on the MALDI Biotarget plate. Once dried, the mass spectra are acquired in the linear positive-ion mode. The image has been created with BioRender.

In conclusion, dilution of milk in double distilled water combined with the super-DHB matrix solubilized ta 10 mg/mL in 70% ethanol provides a one-step lipids fingerprint and unique signatures to discriminate bovine from non-dairy milks and their use as markers for milk adulteration by using routine MALDI-TOF mass spectrometer, which is already used to identify food pathogens and therefore available in many laboratories for food-testing^56^ (Fig. 4). The procedure is rapid, easy-to-use, reproducible and cost effective, which is suitable to qualitatively typify milks and potentially their adulteration.

## Supplementary information

Supplementary Information.
