# Supplementary material for: Discrimination of bovine milk from non-dairy milk by lipids fingerprinting using routine matrix-assisted laser desorption ionization mass spectrometry
Source: Sci Rep. 2020 Mar 20;10:5160. doi: 10.1038/s41598-020-62113-9 (PMC7083858; doi:10.1038/s41598-020-62113-9)
Supplement: Supplementary file 1 — Supplementary Information. [file 41598_2020_62113_MOESM1_ESM.docx]

**Discrimination of bovine milk from non-dairy milk by lipids fingerprinting using routine matrix-assisted laser desorption ionization mass spectrometry**

Philippa ENGLAND^1^, Wenhao TANG^2^, Markus KOSTRZEWA^3^, Vahid SHAHREZAEI^2^, and Gerald LARROUY-MAUMUS^1†^

^1^MRC Centre for Molecular Bacteriology and Infection, Department of Life Sciences, Faculty of Natural Sciences, Imperial College London, London, SW7 2AZ, UK

^2^Department of Mathematics, Imperial College London, London, United Kingdom

^3^Bruker Daltonik GmbH, Bremen, Germany

**
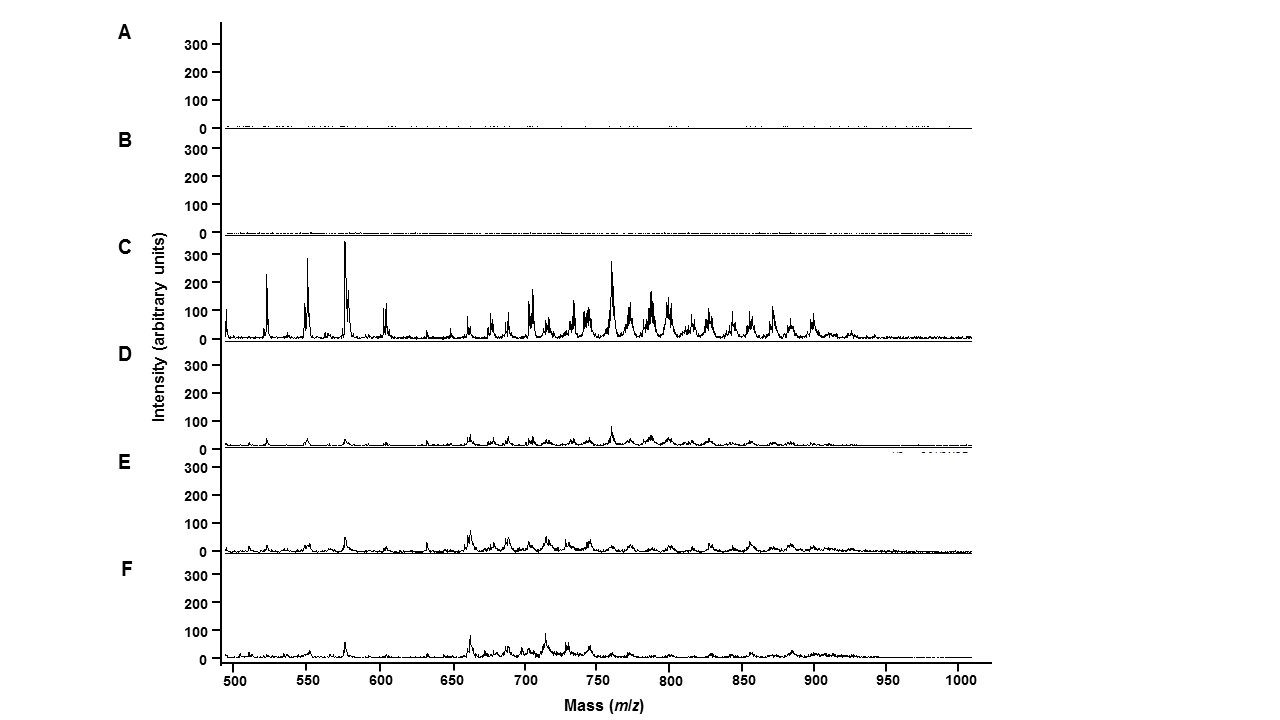
**

**Figure S1:** MALDI-TOF positive ion mode mass spectra of whole bovine milk, undiluted (**A**), 1:2 diluted in ddH_2_O (**B**), 1:4 diluted in ddH_2_O (**C**), 1:6 diluted in ddH_2_O (**D**), 1:10 diluted in ddH_2_O (**E**), 1:20 diluted in ddH_2_O (**F**).

**
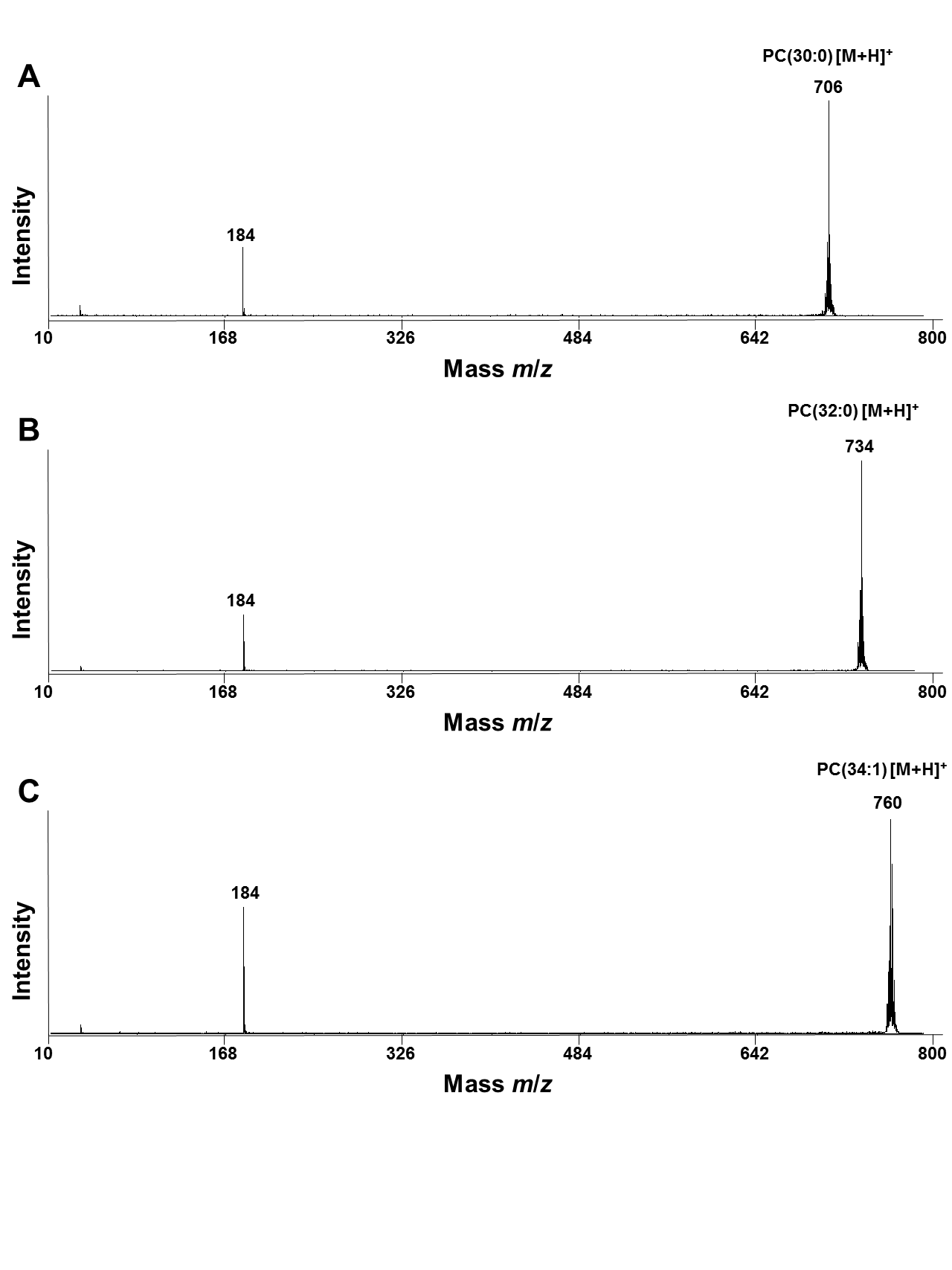
**

**Figure S2**: MS/MS mass spectra of peaks at *m*/*z* 706 (**A**), *m*/z 734 (**B**) and *m*/*z* 760 (**C**) from bovine milk. The fragment at *m*/*z* 184 corresponds to the polar head group fragment assigned to as phosphocholine.

**
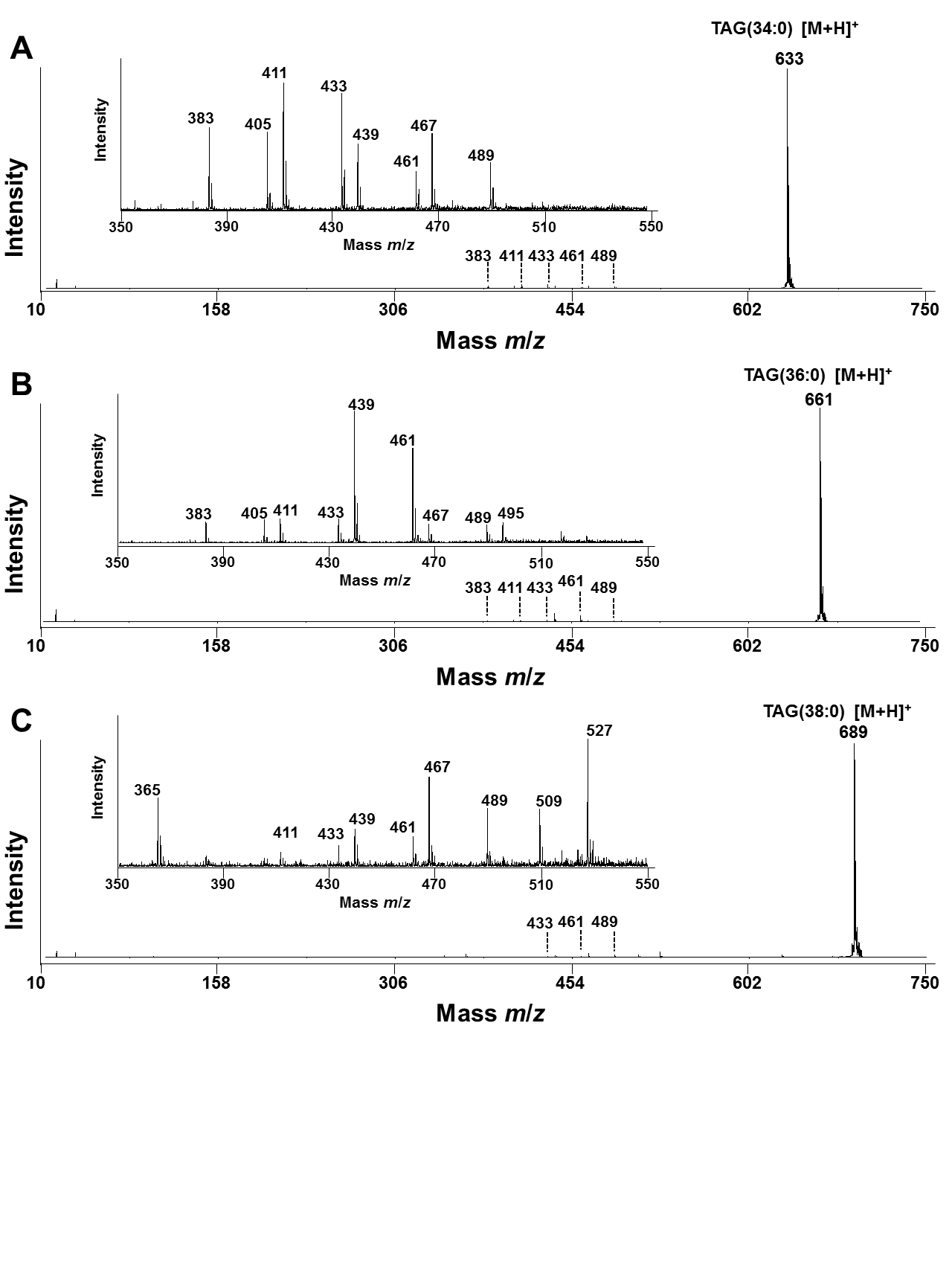
**

**Figure S3**: MS/MS mass spectra of peaks at *m*/*z* 633 (**A**), *m*/z 661 (**B**) and *m*/*z* 689 (**C**) from coconut milk. Insets represents the mass range *m*/*z* 350 to *m*/*z* 550.

| **Lipid species** | **Measured mass (m/z)** | **Ion type** | **Corresponding DAG** | **Residue loss** |
| --- | --- | --- | --- | --- |
|  |  |  |  |  |
| TAG 34:0 | 633 |  |  |  |
|  |  |  |  |  |
|  | 383 | C ion | 20:0 | 14:0 |
|  | 411 | C ion | 22:0 | 12:0 |
|  | 433 | B ion | 22:0 | 12:0 |
|  | 461 | B ion | 24:0 | 10:0 |
|  | 489 | B ion | 26:0 | 8:0 |
|  |  |  |  |  |
| TAG 36:0 | 661 |  |  |  |
|  |  |  |  |  |
|  | 383 | C ion | 20:0 | 16:0 |
|  | 411 | C ion | 22:0 | 14:0 |
|  | 433 | B ion | 22:0 | 14:0 |
|  | 461 | B ion | 24:0 | 12:0 |
|  | 489 | B ion | 26:0 | 10:0 |
|  |  |  |  |  |
| TAG 38:0 | 689 |  |  |  |
|  |  |  |  |  |
|  | 433 | B ion | 22:0 | 16:0 |
|  | 461 | B ion | 24:0 | 14:0 |
|  | 489 | B ion | 26:0 | 12:0 |

**Table S1**: Diacylglycerols formed after fragmentation of the triacylglycerols 34:0, 36:0 and 38:0 from coconut milk by MALDI-ToF MS/MS.


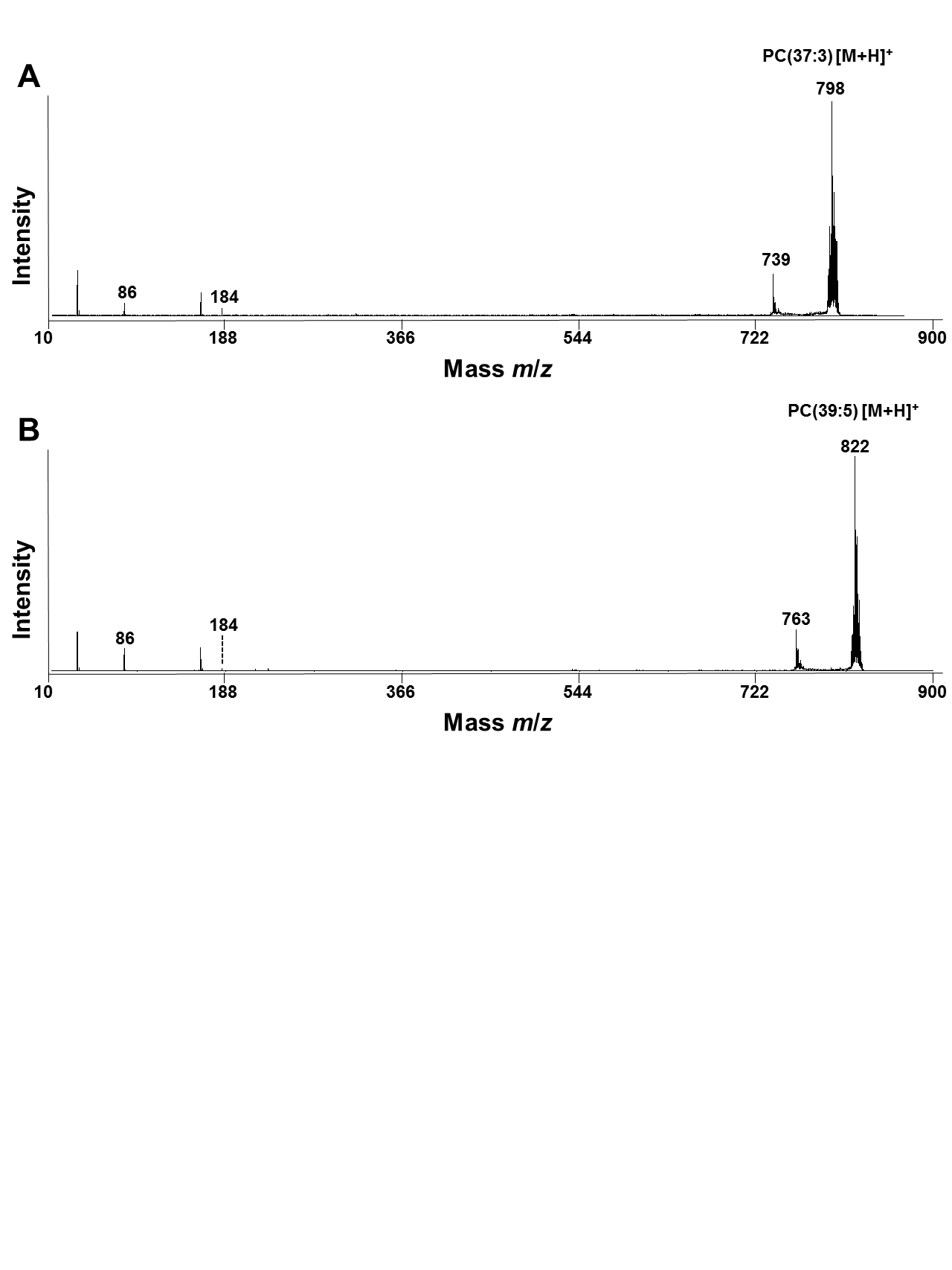


**Figure S4**: MS/MS mass spectra of peaks at *m*/*z* 798 (**A**) and *m*/z 822 (**B**) from soya milk. The fragment at *m*/*z* 184 corresponds to the polar head group fragment assigned to as phosphocholine. The ion at m/z 86 is a characteristic fragment of the choline head group. Neutral loss of the trimethylamine from the phosphocholine head group generate the fragment ion [M+H-59]^+^ at *m*/*z* 739 and *m*/*z* 763 from the parental ion at *m*/*z* 798 (**A**) and *m*/z 822 (**B**) respectively.


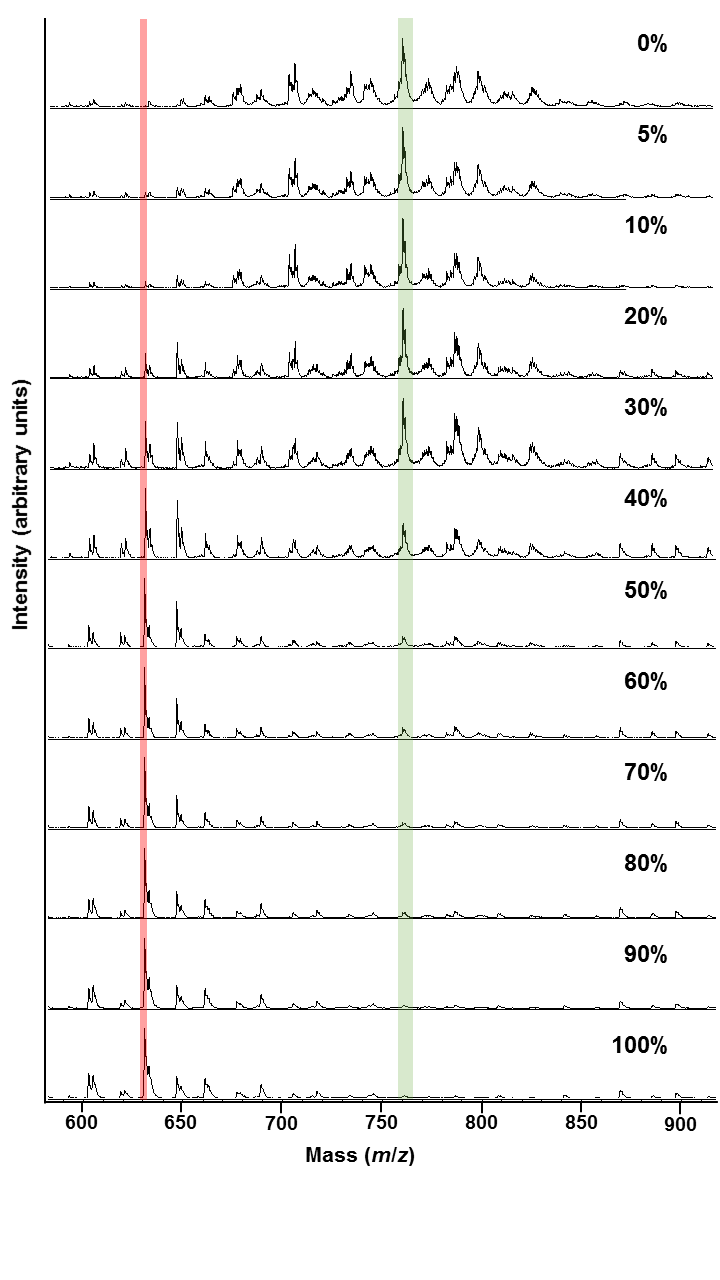


**Figure S5**: Intact lipids fingerprint allows detection of coconut and bovine adulteration. MALDI mass spectra obtained from mixture of bovine milk adulterated at 5%, 10%, 20%, 30%, 40%, 50%, 60%, 70%, 80% and 90% with coconut milk. The reference peak of bovine milk at *m*/*z* 760.5 is highlighted in green and the reference peak of coconut milk at *m*/*z* 633.5 is highlighted in red.


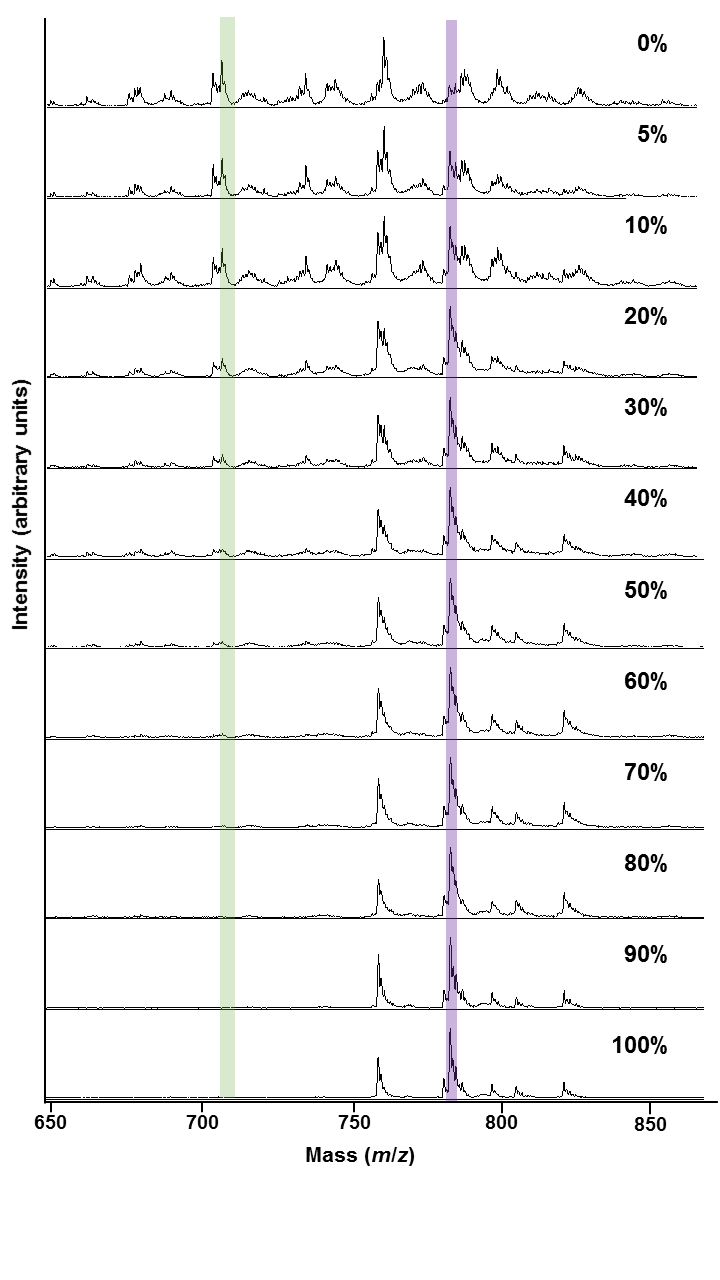


**Figure S6**: Intact lipids fingerprint allows detection of soya and bovine adulteration. MALDI mass spectra obtained from mixture of bovine milk adulterated at 5%, 10%, 20%, 30%, 40%, 50%, 60%, 70%, 80% and 90% with soya milk. The reference peak of bovine milk at *m*/*z* 706.5 is highlighted in green and the reference peak of soya milk at *m*/*z* 784.6 is highlighted in purple.
